# Formative research to develop a school-based, community-linked physical activity role model programme for girls: CHoosing Active Role Models to INspire Girls (CHARMING)

**DOI:** 10.1186/s12889-019-6741-1

**Published:** 2019-04-25

**Authors:** Kelly Morgan, Jordan Van Godwin, Kirsty Darwent, Alison Fildes

**Affiliations:** 1 DECIPHer, School of Social Sciences, Cardiff University, Cardiff, UK; 2 Nursing, Midwifery and Allied Health Professions Research Unit, Faculty of Health Sciences and Sport, Stirling University, Stirling, Scotland; 3 Faculty of Medicine and Health, School of Psychology, University of Leeds, Leeds, UK

**Keywords:** Multi-component, Physical activity, Preadolescent, School, Community, Role modelling, Design, Intervention, Primary school

## Abstract

**Background:**

Physical inactivity is a persistent challenge among girls. School-based physical activity (PA) interventions have shown mixed effects on girl’s activity levels, with multi-component approaches involving both school and community links appearing more effective for sustainable change. The purpose of the current research was to gather views from preadolescent girls, parents, teachers and stakeholders in order to co-produce a multi-component school-based, community linked PA intervention programme.

**Methods:**

Focus groups were conducted in two primary schools with 34 girls aged 9–11 years and 11 parents (10 female, 1 male). In-depth interviews were conducted with four female teachers (including two head teachers). Focus groups and interviews focused on programme design (structure, content and delivery) and potential factors affecting intervention uptake and continued PA participation. A series of stakeholder engagement events occurred throughout the study period. All data were transcribed verbatim and thematically analysed in NVivo 11.

**Results:**

Girls reported that fun taster sessions delivered by role models would encourage them to participate in a school-based role model programme, with tailored taster sessions each week to enhance continued PA participation. Parents and teachers identified a number of barriers to uptake and continued PA participation, and active involvement of stakeholders facilitated the development of intervention strategies. Strategies included; single-sex after-school sessions, use of female role models, low-cost activity options and mapping community provision. Analyses revealed the importance of tailoring the programme to align with local needs, demands and provision.

**Conclusions:**

Data show numerous barriers to intervention uptake and continued PA participation when designing a school-based, community-linked intervention. Adopting a co-production approach, this formative work highlights a number of potential strategies for overcoming these barriers. Findings from the research directed the development and implementation of the CHARMING role model intervention and informed the creation of an intervention logic model.

**Electronic supplementary material:**

The online version of this article (10.1186/s12889-019-6741-1) contains supplementary material, which is available to authorized users.

## Background

Globally, an estimated 3.2 million deaths are attributed to physical inactivity, placing physical inactivity as the 4th leading cause of death worldwide [1]. Alongside adverse physiological outcomes such as increased risk of being overweight or obese, and developing type 2 diabetes [2], physical inactivity is also associated with poor mental wellbeing [3]. With three in four mental illnesses starting before age 15 years [4] and over 80% of adolescents currently insufficiently active [5], there is an urgent need to implement effective strategies to increase physical activity (PA) before PA-related health disparities become entrenched. Adolescent girls in particular have low PA levels, displaying a notable decline during the transition period from childhood to adolescence [6]. As a result, girls tend to be far less active than boys of the same age, a trend observed worldwide [7].

Previous work has shown confusion surrounding children’s perceptions of PA requirements [8]. Among a sample of English preadolescents aged 8–12 years, children were able to identify foods comprising a healthy diet, yet they had limited knowledge of PA recommendations [8]. Preadolescence therefore presents a window of opportunity to intervene on both exposures (e.g., unhealthy foods) and behaviours (e.g., sedentary behaviours) that can have lasting health effects [9] and educate children on healthy lifestyles [10]. A large number of PA interventions include health education as an intervention component; however, one review highlighted the uncertainty about how children’s knowledge of (or beliefs and attitudes towards) PA affects their PA participation [11]. Supporting this assertion, a review on the effectiveness of classroom-based education [12] found increases in general health knowledge, exercise-related knowledge, and self-efficacy about exercise lead to limited success in increasing time children spent in PA outside the school setting. To our knowledge, no intervention to date has attempted to deliver health education messages using PA as the mode of delivery, presenting a potential opportunity to bridge the gap between health education and health behaviours.

Schools have become a popular setting for intervention delivery [13]. A recent systematic review [14] examining the impact of school-based PA interventions targeting older girls (e.g. adolescents) highlighted that multi-component interventions underpinned by theory were more likely to be effective. Such findings echo recommendations outlined within the Medical Research Council [MRC] framework when developing complex interventions [15], emphasising the development stage as key. The importance of co-producing health-behaviour interventions with the target audience [16, 17] has been recognised as one strategy to enhance intervention optimisation [18–21]. To date however, interventions have typically adopted a top-down approach, whereby a researcher engages with the target audience for feedback on developed elements [16] as opposed to undertaking formative work with the target audience during the pre-trial stage [17]. Co-production, a means of involving the target audience in both the design and implementation stages of an intervention, has shown a greater degree of intervention engagement [18], providing the opportunity to co-create a contextually appropriate intervention design. Wight and colleagues [19] emphasise the value of co-production in maximising the likelihood of intervention effectiveness by improving intervention fit with considerations for the target audience perceived needs and acceptability, practicality, intervention theory, and implementation and uptake by stakeholders. Alongside the involvement of a target population, it is also important to engage their gatekeepers (e.g., schools, teachers and parents) in addressing contextual complexities [20]. The necessity of stakeholder involvement throughout intervention design and implementation has been recognised as a crucial element for the facilitation of long-term improvements in PA, sedentary and dietary behaviours [21].

With a specific focus on increasing PA amongst girls, research has shown the importance of providing a wide range of appealing activities [22], providing girl-only sessions [23, 24] and underpinning an intervention framework with theory [15]. Recognising the importance of motivation for the long-term adoption of a behaviour [25], many interventions focus on attempts to promote action by converting motivation into action [26]. Such motivation can come from observing positive results in others and it has been suggested that role models can provide inspiration for young people to become involved in, or maintain involvement in, sport and PA [27]. The World Health Organisation (WHO) specifically recommends the use of role models within local communities to increase PA among females [28]. These recommendations also highlight the importance of ensuring sustainable engagement in PA through the development of school and community links. Interventions linking the school and wider community have shown to be effective in sustaining changes in PA levels among adolescents [11, 29], which ultimately requires effective partnership working within communities. That said, previous studies exploring perceptions of PA role models or school-based intervention designs are limited to adolescent populations and are manly US-based. There is a need therefore to design and implement preventative strategies to help evade the age-related decline in PA that is characteristic of adolescent girls.

To address the multiple level factors that can determine PA, a comprehensive approach is needed for the design of effective interventions. One approach is offered by Bronfenbrenner’s socio-ecological model [30, 31], which emphasises that an intervention should not only focus on the intrapersonal factors (for example, self-efficacy) but on the multiple layers which influence behaviour (for example, visibility of role models and PA community opportunities). To fully understand health behaviours and the contexts in which they occur, the socioecological model highlights the importance of gaining a full appreciation for interrelationships between individuals and the social, physical and policy environment, acknowledging that self-regulation is difficult to establish without wider social and institutional support [32]. Self-determination theory (SDT) has also been widely applied to school-aged children offering an effective framework for evaluating children’s PA behaviours at individual and contextual levels [33]. Highlighting the strength of the SDT in predicting PA, previous studies suggest that while the correlates of PA can be examined from a socioecological standpoint, SDT can be used to address psychosocial influences [34]. Approaches which are based on an empirical understanding of psychosocial factors underlying the target audience’s decision have been shown to increase their likeliness of being effective [35]. Theory integration enables the incorporation of multiple key constructs across theories in order to enhance our understanding of the underlying mechanisms of health behaviour change such as PA. Subsequently, Zhang and Solmon [33] suggest integrating SDT with the social ecological model to harness the strength of each theory.

Providing girls with active role models and links to existing community activities could positively influence girls PA levels before the anticipated decline in adolescence. This paper reports the results of formative research to inform the development and design of a school-based PA role model programme. The theoretical framework for the intervention design integrates SDT and the socio-ecological model. Gathering child, parent, teacher and wider stakeholder perspectives, we set out to: i) seek input on programme structure, content and delivery; ii) identify barriers and facilitators to intervention uptake and continued PA participation; and iii) use these data to subsequently inform the design of the first UK-based tailored PA intervention programme, CHARMING (CHoosing Active Role Models to INspire Girls), with the production of a logic model.

## Methods

### Participants and recruitment

Data collection took place in May 2016. Two primary schools in areas of high deprivation in South Wales (the percentage of children eligible for free school meals exceeded the national average), United Kingdom, were purposively recruited via a Healthy Schools Coordinator. One school was located in an urban region and had a high proportion of Black and Minority Ethnic (BME) pupils (above 87%), the other school was located in a rural area and had a predominant white population (BME population below 12%). All Year 5 (9–10 years old) and Year 6 girls (10–11 years old) and their parents were invited to participate in the study. Parents were given the opportunity to opt their daughter out of focus group participation. Two teachers (including the head teachers) from each school were also invited to take part in an interview. The school authority, children, parents and teachers provided written informed consent before participating within the study. Of the invited 83 girls (across both schools), three girls were opted out by their parents (at the urban school) and 34 took part in the current study. Full approval for this study was given by the Social Sciences Research Ethics Committee at Cardiff University (SREC/1816).

### Procedures

Each school had two child focus groups, which consisted of one ‘active’ group and one ‘inactive’ group as identified by the teacher, with a subsample of children (*n* = 34) randomly selected. Within the rural school, the teacher requested that all children (*n* = 18) be given the opportunity to participate due to the small school size. The rationale for splitting the groups according to their activity status was based on previous research showing that children are more likely to contribute to discussions if homogenously grouped in terms of their PA levels [36]. Both sets of focus groups were broadly representative of the demographics of the school populations. All girls were provided with a study information sheet prior to the study commencement date. Focus groups lasted between 36 and 47 min and interactive activities were used to encourage the girls to contribute ideas to the intervention design. The topic guide questions centred on identifying role models and their characteristics, and the intervention programme design (see Additional file 1).

Parents received a study invitation letter and information sheet, inviting them to participate in a focus group at the school premises. In total, 11 parents (comprising 10 females) took part (8 at the rural school and 3 at the urban school), with focus groups lasting 27–54 min. In the urban school focus group, two parents were from a white British background, with one parent from a minority ethnic background. In the rural school, all participants were from a white British background. A semi-structured topic guide covered the following areas: 1) barriers and facilitators to uptake (i.e., attendance at school sessions) and continued PA participation (i.e., attendance at community clubs), 2) programme logistics and 3) current community provision of PA (see Additional file 2).

At each school, the head teacher and one other teacher (recommended by the head teacher) were invited to participate in a face-to-face interview. A semi-structured topic guide focused on: programme design and delivery, and barriers and facilitators to uptake and continued PA participation (see Additional file 3). Prompts were utilised by the interviewer in the event that open questions did not yield sufficient information and interviews lasted 27–55 min.

The first and second author facilitated all focus groups and the second author conducted all interviews. All participants were reminded that they could withdraw from the study at any time point and that recordings would remain confidential. The research team whom have expertise in qualitative research and intervention design, developed topic guide questions. All focus groups and interviews were digitally recorded and transcribed verbatim for analysis.

A series of face-to-face stakeholder meetings took place throughout the intervention design period involving school-, local authority-, policy- and national governing representatives. Initial meetings provided an opportunity to introduce the study aims and scope current PA initiatives within schools and local areas. Between meetings, communications were maintained via email correspondence. Following the preliminary analyses of focus group and interview data, stakeholders were invited to attend a workshop with discussions centred on: sourcing role models, identifying existing community clubs and activities and avenues for disseminating information on community PA provision.

This collaborative approach is in line with other PA intervention development (e.g. CHANGE! [37], TAAG [38]), with formative research providing a platform to understand the needs, interests and attributes of communities prior to intervention design and implementation. Adopting a co-production approach, the design of the CHARMING intervention incorporated study data alongside stakeholder practice-expertise and knowledge to produce an intervention logic model.

### Data analysis

Focus group and interview data were thematically analysed [39] to allow an exploration of the similarities and differences across datasets. The process involved reading and rereading transcripts to enable the development of a coding framework (using NVivo, Version 11). One researcher coded all transcripts, with 30% of transcripts double coded by a second. Data were grouped and summarised into themes. Any discrepancies in the coding or reduction process were discussed until consensus was reached. Finally, both members of the primary research team selected indicative quotes representing the core of wider views. Field notes and email correspondence were also recorded, filed and included within analyses.

## Results

**Fig. 1:**
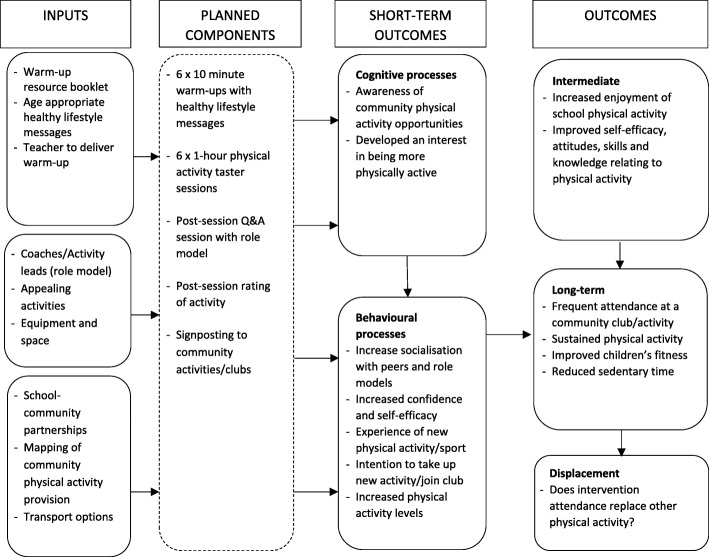
Intervention logic model (Design phase)

The section below is structured as follows: Firstly, results concerning the intervention design are summarised with Table 1 providing an overview of the main intervention components with exemplary quotes. These findings are mapped onto the intervention logic model (Fig. 1). Second, findings concerning intervention uptake and continued PA participation are presented according to each specific domain of the socio-ecological framework, with barriers and future strategies aligned (key themes and exemplary quotes denoted in Tables 2 and 3).

**Table 1:** Quotes regarding intervention design

	Key Themes	Quotes
Structure	Taster sessions	“Maybe have lots of different activities there just in case someone gets bored or something and they don’t want to do that, and they want to do something or maybe they have different things each week.” [Child, Rural school] “Yes, but each week you could do a different thing though.” [Child, Rural school] “...drama the next day, dancing, music, art mix it up, because if you do the same thing every day it’s going to get boring” [Child, Rural school]
	After-school	“I think you’d probably have to do it straight after school, 3.20 to 4.20…the clubs that we do run…seem to run okay in that session…” [Teacher, Urban school] “After school.” [Child, Urban school] “This is where you want to be careful because the majority of pupils, 5 o'clock they go to the Mosque and they finish at seven so it would either have to be straight from school or possibly on the weekend.” [Parent, Urban school]
	Single-sex sessions	“They’ve just done multi-sports and we had lots of take up and the girls have actually really, really enjoyed it and said how they like being involved in a club where there are not so many boys…” [Head teacher, Rural school] “No, only girls.” [Child, Urban school] “Sometimes mixed, sometimes only girls.” [Child, Urban school]
Content	Fun	“By sometimes playing like little mini games.” [Child, Urban school] “Letting us try new things.” [Child, Urban school] “Maybe they could make a little bit of their own game up.” [Child, Rural school] “Maybe we could have kits?” [Child, Rural school]
	Competitive	“To see how good you are and see how much you need to improve?” [Child, Rural school] “So you can see who is the best.” [Child, Rural school] “Then you can show your skills.” [Child, Urban school] “…there are not many dance groups out there that do it for fun. It is too competitive.” [Parent, Urban school] “Keep us more active.” [Child, Rural school] “You might lose at competition.” [Child, Urban school]
	Appealing activities	“Something a little bit different, obstacle courses.” [Child, Urban school] “We won’t be able to play curbsy because we got no curbs and we can only do that on the road.” [Child, Urban school] “And it’s actually finding what they are interested in.” [Head teacher, Urban school] “It would be quite nice if you were going to do something for girls that it wasn't just about falling back on dance [laughs], you know, dance or aerobics which is the other thing which is a wildly boring thing to teach a child.” [Parent, Urban school] “Maybe like tennis or badminton, something a bit different.” [Parent, Rural school]
	Key themes	Quotes
Content	Health messages	“…as long as it was all there and the resources were there and we didn’t have to provide them for ourselves.” [Teacher, Rural school] “…in a way, those free school meal children are at an advantage, because you know they’re getting the right nutrition. They’re not eating rubbish whilst they’re in school.” [Head Teacher, Urban school] “…factors to bear in mind for this age group in particular include how interventions are delivered. With more effective interventions using methods such as peer mentoring, rather than what can be perceived as ‘preachy’ type approaches.” [Senior Researcher, Policy department] “It sounds like the programme would fit in beautifully with our Healthy Schools. We are a Healthy School. So that sounds yes, like something they would understand and participate in.” [Head teacher, Urban school]
Delivery	Female role models	“Because say like you have a problem like, in the body section, girls understand more if it's only girls in the club.” [Child, Urban school] “I don't know why, but I don't feel comfortable with male teachers.” [Child, Urban school] “The only teachers we got in our school is girls, we got no boy teachers.” [Child, Rural school] “I think you would have trouble with after school clubs for girls if there weren’t women running the sessions for all sorts of reasons, but I don't know.” [Parent, Urban school] “So from our point of view, for our Muslim parents to agree to the girls coming, it would probably need to be a female instructor.” [Head teacher, Urban school] “…if someone was my role model, I don't want them to be boring, I want them to be fun.” [Child, Urban school] “…it doesn’t matter if they’re famous or not… because they are who they are…because we think they’re role models, not other people.” [Child, Rural school]
	Links to community opportunities	“I think if we had something on our doorstep, I think they would go. I definitely think they would get more involved…we’ve got parents that are you know… willing to take up opportunities with children, whenever we have things here, if we have events here like after school…” [Head teacher, Rural school]. “I think really it’s having an increase in opportunities for them to try a different variety of sports beyond the school really.” [Head teacher, Rural school]

**Table 2:** Barriers to intervention uptake

Theoretical lens	Barriers*	Quotes	Intervention strategies
Intra	Prior commitments Motivation	“If they were doing another activity.” [Parent, Urban school] “One minute they're all for it, the next minute 'Oh I am not doing that today, I don't want to.” [Parent, Rural school]	• Straight after-school hours • Engaging activities
Inter	Parental interest Family logistics	“I think it would be selling the programme to the parents. I think we need to you know, get the parents involved from the beginning…” [Deputy head teacher, Urban school] “I've got the younger ones, they have to be in bed by eight so as long as we were home by about seven thirty at the very latest so that they could be fed, bathed then bed.” [Parent, Urban school]	• Opt-out consent • Sessions run straight from school
Organisational	Inadequate provision Lack of facilities Lack of staff Cost of activities	“A lot of parents have complained to the school because initially, years ago, I'd say about three years ago, there was a lot of after school activities and they've all just phased out.” [Parent, Urban school] “There isn't a lot of space in this school for sports so a lot of sports really have to be outside if you want to play with any number of people really.” [Parent, Urban school] “I think they’d like a lot of sport after school, but with such a small school … there’s only five members of staff five days of the week, so we can’t offer that.” [Teacher, Rural school] “we haven’t got the capacity if you like to do any more clubs…that would mean buying in somebody else to do that and then that would be the cost implication for them, no matter how much.” [Head Teacher, Rural School] “Would we have to pay to come to the club?” [Child, Rural School] “…it's just the cost of it. That would be the only problem, wouldn't it, really?” [Parent, Rural school]	• Maximum of 30 per session • Minimal requirement of staff time • Sessions run straight from school • Low cost/ free sessions
Community	Role model nature	“I think the barriers are finding somebody to come in and do it. Finding somebody who knows the nature of the culture that the girls are coming from and can you know, not offend anybody, make sure that they’re on side with the parents.” [Head teacher, Rural school]	• Provide parents with role model information
Societal	Cultural perceptions Cultural commitments	“I think you will find it difficult in this school because a lot of the girls are not allowed to take part in these things, whereas the boys are.” [Parent, Urban school] “Because you need to get ready for mosque.” [Child, Urban school]	• Provide single-sex sessions • Appropriate activities • Straight after-school hours

Key themes* presented alongside the Socio-ecological model

**Table 3:** Barriers to continued PA participation

Theoretical lens	Barriers*	Quotes	Intervention strategies
Intra	-		
Inter	Family resource Family logistics	“We have a lot of new families that are coming in to the country who are still going through the process of actually getting their funding sorted or finding a job.” [Teacher, Urban School] “So, especially when you've got more than one child, I've only got the one so swimming was easy for me, but that was like fifty-odd pound for a term, so if you've got like three, four kids...” [Parent, Rural school]	• Low cost options
Organisational	Awareness of opportunities	“I’m not aware… well aware of what’s available in the community …” [Deputy Head Teacher, Urban School] “There may well be things out there. But here, I’m not aware of what I can and can’t have, and it may be something as simple as communicating.” [Head teacher, Rural school]	• Mapping community provision • Engage key community groups and leaders
Community	Unreliable transportation Lack of opportunities Mixed-sessions Cost of activities Club opening hours	“Geographically, we’re not best placed here. … We need to take a bus to the local pool and most clubs… are quite a distance from here .” [Head teacher, Rural School] “…if it was four o’clock and it was up in [local area], sometimes we don't have a bus until ten to four, because it stops.” [Parent, Rural school] “I’d say in this area, there isn’t much.” [Teacher, Rural School] “…our comprehensive school is closing and that’s got a leisure centre attached to it. That’s our nearest facility here.” [Head teacher, Rural school] “… but from what the children tell us they do, I don’t believe there can be a lot.” [Deputy Head Teacher, Urban School] “…we wanted something that would encourage those that were put off by maybe what they saw as more aggressive actions of the boys when they’re playing… their competitive edge comes out and it puts the girls off.” [Deputy Head Teacher, Urban School] “It’s maybe not at an appropriate time or it costs.” [Deputy Head Teacher, Urban School] “I think there’s possibly things available for them but for our girls it’s obviously quite difficult to access because of time constraints really.” [Head Teacher, Urban school]	• Mapping community provision • Options within close proximity or linked to local transport • Source single-sex opportunities • After-school hours
Societal	Cultural perceptions	“They’re in the Mosque at five and they’re there till seven o clock. So obviously accessing sports facilities is quite difficult for them and it’s not necessarily part of their culture really to do sporting activities after school.” [Head Teacher, Urban school]	• Challenging cultural norms

Key themes* presented alongside the Socio-ecological model

### Intervention design

#### Structure

1. *Taster sessions*

The majority of girls wanted the opportunity to experience a different session each week. The girls reported that they might become bored of having the same activity each week and would possibly be less likely to attend.

2) *After-school*

Based on their experience of delivering extra-curricular activities, teachers at both schools indicated that directly after school was an appropriate time for delivering the intervention. Within the rural school, teachers also suggested the last session during the school day would be a feasible option to guarantee intervention attendance. Parents in both schools agreed that after-school on the same day each week would be best, as it would allow them to plan accordingly. Within the urban school however, both children, parents and teachers identified religious activities as a potential barrier to uptake. The girls provided a range of responses (e.g. lunchtime, before school, after school, weekends) but when asked for a show of hands, a large majority preferred sessions to be delivered straight after school.

3) *Single-sex sessions*

The inclusion of single-sex sessions was discussed among girls and the majority expressed a desire for girl-only sessions. This point was further emphasised by teachers within the urban school who highlighted that they had observed a greater uptake of after-school clubs by girls when there were less boys in attendance. The notion of single-sex sessions also became apparent when discussing barriers to intervention uptake and cultural beliefs.

#### Content

1. *Fun*

The girls were keen to ensure that the sessions were fun. The facilitator asked for ideas of how to achieve this and the girls provided a range of suggestions including; trying a new PA each week, learning new skills and tricks, playing games and having new sports kit.

2) *Competitive*

The majority of girls reported they would like the sessions to contain an element of competition. A number of girls felt it provided an opportunity to demonstrate skills and test themselves. A few girls thought sessions should not involve competition, with one girl revealing a fear of losing. In discussions, a parent also shared her preference for non-competitive sessions, drawing on her previous difficulty of trying to locate a non-competitive community dance group while one head teacher suggested the inclusion of boys within sessions made the sessions too competitive.

3) *Appealing activities*

With an aim to maximise intervention uptake and attendance, girls were asked what activities they would like to take part in. A range of activities were suggested including; traditional team sports (e.g. football, netball, hockey, and volleyball), individual sports (e.g. gymnastics, golf, martial arts, darts) general PAs (e.g. climbing, dance) and alternative options (e.g. dodgeball, obstacle courses). Girls also suggested non-active sessions such as photography, arts and cooking. Parents identified a range of activities also, covering many of the activities suggested by the girls. Some parents were keen for the children to ‘try something different’ with ‘fighting activities’ specifically discouraged in the rural focus group.

4) *Health messages*

From a practical perspective, teachers at both schools did not perceive any barriers to the delivery of health messages. They were willing to deliver the messages as a component of the intervention on the basis that all related-resources were provided.

When discussing the types of messages to be delivered, the researcher gave examples relating to dietary intake, water consumption and PA recommendations. One teacher stated how this intervention component would fit well with the school’s current provision as a member of the ‘Healthy Schools’ network (a network of schools which actively promote, protect and embed the physical, mental and social health and well-being of its community through positive action [40]). Contrastingly, one head teacher expressed an urgent need to tackle unhealthy eating practices within their school, particularly among children not receiving school lunches as both excessive amounts of food and unhealthy food items mainly comprised children’s packed lunches.

#### Delivery

1. *Female role models*

Girls identified a range of role models, which included family members, friends, athletes and celebrities. When asked whom they would like to lead the PA sessions, across both schools there was a mixed response, with suggestions of family members, friends and new people. Some of the girls at the urban school described how they would be more comfortable with a female leading the session, providing explanations related to preferences for female teachers/mentors when dealing with body issues. In the rural school, one girl alluded to the fact that they had only female teachers at their school. This theme re-surfaced when discussing the potential barriers to intervention uptake and continued PA participation. Girls also identified a number of characteristics to describe role models, comprising mainly interpersonal traits such as; ‘fun’, ‘kind’, ‘friendly’, ‘helpful’, ‘honest’ ‘confident’ and ‘supportive’.

2) *Links to local community opportunities*

The opportunity to link the intervention to community activities within close proximity of the school was particularly emphasised by the rural school head teacher. This theme appeared to be entwined with many perceived barriers to continued PA participation and coincided with a lack of community resource and awareness. The importance of linking to existing community activities was also raised among discussions with stakeholders.

#### Barriers

A range of barriers were identified that were perceived to affect intervention uptake and continued PA participation (Table 2 and 3 respectively). Barriers included; 1) prior commitments, 2) cost, 3) family logistics and 4) cultural perceptions. Specific barriers were also pertinent to each school. Each identified barrier will be summarised in relation to the socio-ecological framework.

#### Intra

Girls and parents identified existing commitments as a barrier to intervention uptake (including current PAs and religious practice). Additionally, parents also perceived their daughters’ motivation as a barrier. Both girls, parents and teachers agreed that scheduling the sessions immediately after the school day would be a potential strategy to enhance intervention uptake.

#### Inter

A parent in the urban school highlighted the implications of club closing times and how this impacted upon family-life routines and the likelihood of their child being able to attend community sessions. The deputy head teacher from the urban school stressed the need for engaging parents with the intervention early on, to ensure the programme was well received and to maximise uptake rates.

#### Organisational

Parents and teachers reported four main barriers to intervention uptake rates; cost, current activity provision, school facilities and inadequate staff numbers. Cost was identified as a major barrier by parents and teachers across both schools. One parent implied that the cost of attending sessions at school would be the only barrier affecting girls’ attendance while the urban school head teacher believed that some parents would struggle to pay for community clubs as they had only recently arrived in the UK. Parents identified a number of barriers linked to family logistics such as a childcare provision and additional costs accrued within large families, with some parents highlighting cost implications to allow siblings to attend community sessions also. Within the rural school parents thought that the school lacked extra-curricular sporting opportunities, highlighting that provision had dramatically reduced over previous years. In conjunction, a teacher within the rural school noted that she was aware that pupils wanted more extra-curricular activities but simply stated there were not enough staff to provide activities, with the head teacher discussing cost implications of greater staffing. A parent in the urban school was also concerned about the availability of space for providing activity sessions, highlighting that the outdoor yard space was the only potential area.

Staff awareness of existing community activities was perceived as a barrier to continued PA participation. At both schools, the head teachers described their lack of awareness of community activities. The rural school head teacher concluded a lack of opportunities based on what the children tell them and the urban school head teacher highlighted the need for greater communication between the school and community organisations.

#### Community

Within the urban school, the head teacher identified the role models delivering the session as a potential barrier and discussed the possible implications of role models entering the school without an awareness of religious practices or an existing relationship with parents. The head teacher stressed the importance of selecting role models who were sensitive to the diverse cultural and religious needs within the school and emphasised this further by suggesting the requirement of providing female role models only.

Across both schools, teachers identified five further barriers that would affect whether girls took part in subsequent community activities. These were; unreliable transport, lack of opportunities, cost, mixed-gendered sessions and club opening hours. A unique barrier to continued PA participation identified by parents and teachers in the rural setting was the school’s location, residing in a rural community with a lack of reliable transport in the area. This barrier was further emphasised by a perceived lack of PA opportunities within the rural community. The head teacher expressed a desire for the girls to access a wide range of opportunities that are not traditionally available in school however, she acknowledged the vast implications of the nearest facility closing down. A unique barrier affecting whether girls attended or not within the urban school was the use of mixed-sex sessions. The head teacher perceived that boys tended to evoke hostile and competitive elements to the sessions and therefore girls would be less likely to enrol and persist with the sessions. At the urban school, the head teacher highlighted the need for community clubs to account for children’s wider commitments when deciding on club operating hours.

#### Societal

Two common perceived barriers to intervention uptake and continued PA participation within the urban school were societal perceptions and religious practice. Parents discussed the acceptability of Muslim girls taking part in sports and spoke of how religious commitments or lack of permission from their families may prevent them attending. This point was also stressed by teachers, alluding to the current lack of involvement of Muslim girls in current extra-curricular PA. On numerous occasions girls talked about their current schedule of attending mosque after school, a point which emphasised by both parents and teachers. Providing sessions straight after school would potentially offer a window for girls to attend the intervention before attending mosque. One teacher questioned whether the provision of food for girls heading straight to the mosque from the activity session was an option.

#### Stakeholder engagement

Overall, seven face-to-face meetings occurred and six stakeholders attended the intervention workshop. Stakeholders’ views echoed study data, highlighting concerns around deprivation and cost, the lack of resources and facilities for schools, parental engagement and religious and cultural influences. Various strategies to overcome cost implications included; use of existing community schemes, utilising voluntary role models and forming links with secondary schools. In relation to barriers concerning religious beliefs, one stakeholder highlighted a common misconception among communities, in that girls-only sessions are often misconstrued as Muslim-only sessions. One strategy to overcome such misconceptions included the engagement of prominent and influential community members during intervention implementation who could raise awareness of the sessions and promote engagement. Discussions also centred on the importance of sourcing role models who have had prior experience of working with the target group. Avenues for disseminating information on community provision to families included the use of: social media platforms (e.g. Facebook, Twitter), existing school communication channels (i.e. newsletters, texting system, and family engagement officers), role models and community leaders. Informed by data analyses and stakeholder discussions, an intervention logic model was devised (Fig. 1).

## Discussion

Reviews and meta-analyses have recently demonstrated larger effects among PA intervention designs which are; girls only, schools-based, multi-component and underpinned by theory [14, 41]. Calls for the inclusion of objective PA measures and greater involvement of preadolescent girls are also documented among reviews [42]. The literature identifies role models as a potential strategy to inspire young girls to become involved in, or maintain involvement in, PA and sport [27], with a recent longitudinal Australian study reporting that older girls were significantly more likely to be active if they had a role model [43]. Despite limited research exploring the use of role models to inspire PA participation and a lack of robust trials, policy recommendations strongly support the use of role models for tackling rising inactivity levels [42, 44]. Previous studies exploring perceptions of PA role models or school-based intervention designs are limited to adolescent populations. In an attempt to fill this understudied gap, the current study designed a school-based role model programme using a participative community approach with the target audience and stakeholders.

The first aim of this study was to gather views from preadolescent girls, parents, teachers and wider stakeholders in order to inform the design of a school-based, community-linked role model programme. Eliciting views on intervention structure, content and delivery, data revealed the importance of incorporating multiple viewpoints. Girls were clear that they wanted sessions to be fun and contain an element of competition. This finding is consistent with Jago et al. [45] who identified fun as a key motivator for PA among adolescent girls [46]. To ensure the programme is enjoyable, a key feature of the intervention structure is the provision of taster sessions, whereby a different activity is delivered each week. Girls believed that the programme would become boring with the same weekly activity and instead identified a range of appealing activities that they would like to attend. Providing girls with such ownership over the intervention design and ensuring sessions are fun and enjoyable, is consistent with multiple principles of SDT, including intrinsic motivation, relatedness needs, autonomy support and autonomous motivation [25]. In order to cater for the varying needs of a competitive element, each taster session should provide opportunities for competition and participation without an emphasis on winning. Adopted within previous intervention designs [47], this approach acknowledges that experiences of competence vary upon success or failure at challenging physical activities or as a function of feedback from the role model [48]. After-school was identified as the most suitable time to deliver taster sessions, particularly within the urban school. Teachers, parents and children suggested that this timing would enhance their ability to attend the sessions without encroaching on other commitments.

Consistent with other studies [47, 49, 50], data revealed that single-sex classes were deemed most likely to increase both intervention uptake and continued PA participation. The majority of girls reported that they would prefer single-sex sessions and one teacher expressed the perceived benefits, highlighting greater engagement of girls in previous single-sex school initiatives. Similarly, in other studies [49–51], girls reported greater opportunities to develop skills and experience lowered self-consciousness and increased attention from session instructors in single-sex sessions. Camacho-Minano and colleagues [52] stress the importance of girl-only interventions not only to meet the unique needs of girls but also to avoid the typical PA contexts, such as praise for power, strength and aggressiveness [53], which tend to reinforce gender stereotypes and disadvantage girls.

Similarly, when discussing intervention delivery, this theme linked with the suggested requirement of sourcing female role models to lead sessions, with some girls reporting that they would feel more comfortable and have greater relatability about body concerns with female leads. Parents and teachers in the urban school also encouraged the use of only female role models, highlighting the complexities surrounding religious beliefs. Girls identified a range of characteristics that could inform the recruitment of role models, with interpersonal traits such as fun and friendly and behavioural traits such as helpful and kind deemed most important. Throughout discussions to identify role models, perceptions of personal connection were eminent, with findings directly underpinned by the relatedness principle of SDT.

Concerning the inclusion of health messages, teachers agreed that this component would form an important part of the intervention and fit appropriately into the school ethos. Both teachers identified the need to receive support from the research team in order to deliver messages during sessions. Discussions with policy makers highlighted the importance of ensuring messages were age-appropriate, clearly communicated and delivered in a contemporary manner.

The second aim was to examine the perceived barriers to intervention uptake and continued PA participation. Providing a framework for organising and representing key emergent themes, the socioecological model is an effective tool for conceptualising the many factors influencing PA behaviour [54]. Parents and teachers identified a number of barriers while discussions with stakeholders highlighted numerous strategies to help translate findings into practice. Within both schools, cost was identified as an overarching barrier, presenting across multiple domains of the socioecological model. Specifically, costs associated with session attendance (both at school and within the community), including the need for extra staffing and provision for siblings at community clubs, were perceived as significant barriers to both intervention uptake and continued PA participation. Cost is a commonly cited barrier in the literature concerning families from lower socioeconomic backgrounds [55, 56]. Other barriers identified included lack of transportation, timing of community clubs and family logistics, which are also consistent with previous research [37, 54]. Strategies to support children and their families may be required where access to transport for travel to and from community clubs is lacking [57].

Throughout the developmental process, we encountered considerable contextual complexities (e.g. different cultures, school locations, and single-sex staff). Stakeholder engagement was vital to ensure strategies addressed such complexities and that future implementation would reflect cultural contexts [19]. Within the urban school, cultural considerations commonly underpinned discussions and it was very clear that the use of female role models would positively influence the decisions of girls to participate, and most likely parents, to allow participation in a school-based role model programme. At the inter-personal level, parents play a vital role in supporting young children to be active through their attitudes, parenting practices and behaviours [58, 59]. In the current study, parents and teachers were ambivalent as to whether Muslim girls would be allowed to participate in such a PA programme as they considered it to be outside of the norm. Previous studies have detailed how Muslim women following their faith cannot engage in mixed gender sports and how some activities are deemed culturally unacceptable [60]. Research with religious scholars has also highlighted that while being active is supported by Islam, revealing parts of the body, often the case with sportswear, is deemed inappropriate for Muslim girls [61]. It is therefore important to assure parents that children will attend girl-only sessions and be permitted to wear cultural dress for the sessions. Furthermore, providing girls with a choice of activities which differ weekly will maximise the likelihood of participation in sessions. This data further highlighted the importance of tailoring at the local level and considerations for the cultural context of implementation [21]. Future work should examine Muslim girls’ participation in the intervention and or community clubs to gain greater insight in whether social, cultural or environmental factors present the greatest barrier to participation.

To enhance continued PA participation, the intervention should involve clear sign posting to community clubs and ensure school staff are aware of existing opportunities. Building partnerships between schools and community sport and recreation providers will help to facilitate links and future implementation [47]. One strategy is to formally link taster sessions with existing community sport and recreation providers; this will ensure consistency of role models, activities and available opportunities for girls. While exploring the barriers specific to this intervention component, the involvement of key stakeholders in the co-production process was a crucial mechanism for tailoring intervention strategies to the target group and intended delivery setting. The wide-ranging expertise and experience of stakeholders also identified existing initiatives for girls in the communities and provided insights into what has or has not worked well previously.

The third aim was to develop an intervention logic model outlining the design of the CHARMING study. During the co-production process, the research team reflected on the study data and input from all stakeholder engagement events. As emphasised by NICE guidelines [62], the intervention design needs to incorporate sound knowledge on the community needs and plans to build upon existing community resources all the while taking into account the local and national context. Creation of the intervention logic model provides a systematic overview of what the intervention will do, what the intended benefits are for the target population and will now be used to guide future development, implementation and evaluation.

All of the above findings will be used to specifically design and pilot the intervention for this population. Evident strengths of this study design are the triangulation of data (i.e. children, parents and teachers) and stakeholder consultation events. When designing a multi-component intervention that includes both a school and community element, partnership working is crucial and often depends upon mutual interests and investments [47]. While guiding intervention development, consultations with stakeholders aided the development of partnerships between organisations and enabled stakeholders to remain engaged with the project. This process has previously shown more results that are favourable in the long-term, with stakeholders being more likely to have an invested interest and make use of the generated evidence base [63, 64].

Some limitations to this study need to be acknowledged. In line with previous research showing the difficulties in engaging parents from low-socioeconomic areas [29], we encountered a low turnout of parents within the urban school. Prior to recruitment, the school staff had informed the research team of the difficulty they have historically had with engaging parents in school activities. Furthermore, all data were obtained from families living in two different areas within South Wales and no data were gathered from families of higher socioeconomic backgrounds, which limits our ability to generalise to other context or countries. The present study provides insights into how co-production with stakeholders can complement existing intervention development guidelines. While not surfacing in the present study, difficulties can arise when faced with competing priorities among stakeholders, this is a consideration for future intervention implementation and refinement particularly when sourcing community activities.

## Conclusions

Based on the findings of the formative research presented in this study, the CHARMING intervention has been developed along with a logic model to guide subsequent testing and refinement. The intervention aims to increase PA levels and promote sustainable changes among preadolescent girls through role modelling and increasing awareness of opportunities for community PA. Future research must examine the feasibility of recruiting role models and tailoring design and implementation at the local level.

## Additional files

Additional file 1: Child Focus Group Guide. A guide to provide further details of the questions covered within each child focus group. (DOCX 24 kb)

Additional file 2: Parent Focus Group Guide. A guide to provide further details of the questions covered within each parent focus group. (DOCX 20 kb)

Additional file 3: Interview Guide. A guide to provide further details of the questions covered within teacher interviews. (DOCX 20 kb)

## Abbreviations

BME: Black and Minority Ethnic
CHARMING: CHoosing Active Role Models to INspire Girls
NICE: National Institute of Clinical Excellence
PA: Physical Activity
SDT: Self Determination Theory
WHO: World Health Organisation
