# Supplementary material for: Formative research to develop a school-based, community-linked physical activity role model programme for girls: CHoosing Active Role Models to INspire Girls (CHARMING)
Source: BMC Public Health. 2019 Apr 25;19:437. doi: 10.1186/s12889-019-6741-1 (PMC6485173; doi:10.1186/s12889-019-6741-1)
Supplement: Supplementary file 1 — Child Focus Group Guide. A guide to provide further details of the questions covered within each child focus group. (DOCX 24 kb) [file 12889_2019_6741_MOESM1_ESM.docx]

**FOCUS GROUP WITH CHILDREN**

Please tell us your first name and age, explain who lives with you in your home, and just for fun, tell us what you like to do most in your free time.

**WHO ARE ROLE MODELS FOR YOUNG GIRLS?**

Use a white board or flip chart to write the definition of ‘role model’—i.e. a person you respect, follow, look up to or want to be like.

- Who do you think are role models for girls your age?

If we think about people who are active, who like to exercise or like to play sport:

- Is there a person who you think of as your role model for exercise, activities or sport?
  - Are there any famous people you admire or think of as your hero/ role model?
  - Why do you prefer people you know / or famous people?

Discuss in pairs and then feedback:

- What do you like about these people? (I.e. what are the qualities or characteristics of your role model (age, gender, skill, sporting ability and personality?)

(Researcher to write the responses on flipchart- spider diagram getting the whole group to feedback answers).

- As a group which of these are most important for a role model to have? (top 5 qualities)

**INTERVENTION DESIGN**

**“Now we’ve thought about who our role models are and what types of things we like about these role models, we are going to think about some ideas of how role models could come and deliver activity sessions to you at school”**

Children to write activities on post-it notes and for them to stick on board

- What types of activities would you like to take part in?
- Are there any activities which you would like to take part in but you feel you can’t?
- When would you prefer to do exercise/activity/sport?
- Before school/lunch/after school/weekend
- Same day each week? Same time?
- How could role models make the sessions fun?
- Where would you like the sessions to be run? (E.g. indoors or outdoors or mixed?)
- Would you like to try many different activities or do more of the same activity?
- Would you like to take part in more sports competitions? Are there any reasons for that?

**CURRENT PHYSICAL ACTIVITIES**

- What sports or physical activities are you currently involved in? Have you previously played sports or taken part in activities which you no longer do? Why?
- Do you take part in these during school time or after school?
- How do you get to these activities? (transport / which family member)
- When you go to your evening / weekend activities what do your parents usually do? (drop off, stay for session, exercise also)
- What time in the evening / on the weekend do you take part in these activities?
- Do friends in your class/school also go to these activities? / Have you made new friends at these activities or are they friends from your community?
- How do you find out about new clubs / sports / activities in your area?
