# Supplementary material for: Formative research to develop a school-based, community-linked physical activity role model programme for girls: CHoosing Active Role Models to INspire Girls (CHARMING)
Source: BMC Public Health. 2019 Apr 25;19:437. doi: 10.1186/s12889-019-6741-1 (PMC6485173; doi:10.1186/s12889-019-6741-1)
Supplement: Supplementary file 2 — Parent Focus Group Guide. A guide to provide further details of the questions covered within each parent focus group. (DOCX 20 kb) [file 12889_2019_6741_MOESM2_ESM.docx]

**PARENT FOCUS GROUPS**

**Community physical activity opportunities**

We are going to focus on the current physical activity opportunities for your child in your community.

- Does your child currently take part in any before/during/after-school community activities?
- What time do these activities usually take place?
- How do they get to these activities? (transport / which family member)
- What do you do whilst your child is at an evening class? (e.g. wait around, go home, exercise)
- Have they previously played sports or taken part in activities which they no longer do? Why?
- How do you hear about community activities / sports clubs?
- How would you like to hear about new activities for your child?
- Are you aware of local clubs and facilities where your child can take part in physical activities and sports?

**Role Models**

Now thinking about your child’s desire to be physically active:

- Where do you feel your child may get inspiration from to lead an active lifestyle?

- Do you consider yourself or any family member to be an active / sporting role model for your child?
- Do you think your child has any sporting role models?

**Intervention design**

<Provide a brief summary of the proposed intervention>

- When would you prefer your child to take part in the exercise/activity/sport?
- Before school/lunch/after school/weekend
- Same day each week? Same time?
- Are there any barriers to your child attending a new school club directly after school ends?
- What are the main barriers, which would stop your child taking part in an evening activity? (E.g. time, money, no transport, other siblings).
- How far would you be willing to travel to take your child to an activity?
- If there was a new evening club starting in your area and your child wanted to attend, what factors would you first consider before deciding if your child could attend or not?
- Are there any activities you would like / not like them to take part in? Why?
