# Supplementary material for: Formative research to develop a school-based, community-linked physical activity role model programme for girls: CHoosing Active Role Models to INspire Girls (CHARMING)
Source: BMC Public Health. 2019 Apr 25;19:437. doi: 10.1186/s12889-019-6741-1 (PMC6485173; doi:10.1186/s12889-019-6741-1)
Supplement: Supplementary file 3 — Interview Guide. A guide to provide further details of the questions covered within teacher interviews. (DOCX 20 kb) [file 12889_2019_6741_MOESM3_ESM.docx]

**INTERVIEW GUIDE WITH HEADTEACHER/TEACHER**

**Background information**

- How long have you been teaching for?
- How long have you been a member of staff at this school?
- Can you tell me about your current role?
- What previous involvement have you had with the delivery of physical activity in your school?

**1. Physical activity for girls**

- Could you talk about your feelings towards young girls’ ability to access and maintain physical activities in your community?
- Can you identify any barriers / facilitators to accessing and maintaining physical activities?
- Can you tell me what you understand are the main barriers / facilitators preventing / helping young girls from accessing and maintaining physical activities in the later years of school?
- Do you think that schools should promote participation in physical activity?
- What do you think the role of schools is in inspiring young girls to be physically active?
- What are the main challenges facing schools in achieving this?
- Does the school currently have policies and practices to support opportunities for girls to be active? If yes, can you please tell me about them?

*If not mentioned, ask about:*

- Any before/during/after school clubs
- Access to individuals with expertise in activities
- Inter-school competitions
- Do after-school clubs and activities tend to be split for girls and boys?
- Who typically delivers PE classes or after-school activities to the girls?
- Do they have mixed or separate lessons?
- Are clubs available for girls in the community?
- Links with community clubs/sports opportunities
- How does your school learn about after-school community clubs for children?
- How well do you feel the school is linked up with groups / individuals who provide community physical activities / sports?
- Is the school’s location perceived to be an awkward area in relation to community clubs?
- Are there any leisure facilities near the school? (Leisure centres, outdoor pitches, swimming pools…)
- How big is the catchment area of children attending the school?
- How does this effect access to clubs / physical activities?

**2. Intervention**

- When do you feel would be the best time of day to run the role model sessions and why?
- What will be the main barriers to children attending these sessions?
- Can you think of any solutions to these barriers?
- Are there any social networks to continue long-term contact with a role model?
- Do you foresee any barriers to a teacher using the activity resource packs to warm the children up before the session?
- Can you think of any solutions to these barriers?
- Is there anything else you would like to add about the provision of physical activities for young girls or the intervention design more generally?
